# How Pleasant Sounds Promote and Annoying Sounds Impede Health: A Cognitive Approach

**DOI:** 10.3390/ijerph10041439

**Published:** 2013-04-08

**Authors:** Tjeerd C. Andringa, J. Jolie L. Lanser

**Affiliations:** ALICE Institute, Artificial Intelligence, University of Groningen, Broerstraat 4. Groningen 9747 AG, The Netherlands; E-Mail: jjllanser@gmail.com

**Keywords:** quietness, quiet areas, quality-of-life, soundscape, mind-states, pleasure, annoyance, needs, arousal, attention, audition, core affect, motivation, safety

## Abstract

This theoretical paper addresses the cognitive functions via which quiet and in general pleasurable sounds promote and annoying sounds impede health. The article comprises a literature analysis and an interpretation of how the bidirectional influence of appraising the environment and the feelings of the perceiver can be understood in terms of core affect and motivation. This conceptual basis allows the formulation of a detailed cognitive model describing how sonic content, related to indicators of safety and danger, either allows full freedom over mind-states or forces the activation of a vigilance function with associated arousal. The model leads to a number of detailed predictions that can be used to provide existing soundscape approaches with a solid cognitive science foundation that may lead to novel approaches to soundscape design. These will take into account that louder sounds typically contribute to distal situational awareness while subtle environmental sounds provide proximal situational awareness. The role of safety indicators, mediated by proximal situational awareness and subtle sounds, should become more important in future soundscape research.

## 1. Introduction

This theoretical paper approaches the relation between quiet or pleasant areas and sustainable health from a cognitive science point of view. The outcome of this paper is a qualitative cognitive model that explains how the sounds that comprise sonic environments promote or impede health. The core idea of this paper is that quiet and pleasant sonic environments allow the listener full freedom and control over mind-states. In contrast, annoying sounds “force” one to be vigilant or to attend particular sources. This paper therefore interprets pleasantness, and the absence thereof, as an indication of whether we exhibit proactive or reactive behavior. Prolonged presence in annoying sonic environments limits proactive adaptive behavior, which erodes proactive optimization of long-term needs with ensuing health effects.

We propose that the sonic features that facilitate freedom of mind-states comprise audible indications of safety: if basic and evolutionary old perceptual processes find ample safety indicators, they allow the newer and higher centers of the brain (typically the cortex) full freedom to address needs that transcend the here and now proactively. In the absence of safety indicators, attentional resources are continuously on alert to address immediate needs reactively, which corresponds to aroused mind-states and an associated focus on the here and now.

The word “quiet” has different dictionary meanings (here from the New Oxford Dictionary). When used in direct reference to a sound or sound source, “quiet” refers to making little or no noise. When used in reference to a place, period of time, or situation it means without much activity, disturbance, or excitement. The third meaning of “quiet” refers to mind states that are not disturbed or interrupted: “*He wanted a quiet drink to contemplate his life*”. “Tranquil” is a synonym for “quiet” in the second and third meaning. 

It is useful to describe these different meanings of quietness in some detail. The first meaning is one in which quietness can be measured as sound levels. This is well within our current technical ability (e.g., [1]) and forms the basis of current policies. This interpretation is *not* addressed in this paper. 

The second meaning of “quiet” refers not to sounds *per se*, but to the interpretation of places, periods, and situations as one of perceived inactivity or the absence of disturbances and excitement. This is indicative of the continuation of normalcy, the absence of pressing situational demands, and therefore of safety. This second meaning can be emulated by future measurement systems that produce similar appraisals of the state of the world as human interpreters do. 

The third meaning of “quiet” refers to prolonged and/or uninterrupted mind-states. This is a locus-of-control issue: whether control over the content and activities of the mind reside with the individual or the environment. This third variant of the concept of quietness can be estimated by a cognitive model describing which (and how) perceptual events claim control over mental-states. We will present such a model in Section 3. 

This paper connects the theoretical foundations of the concepts of a quiet state of the world (second meaning) and a quiet, or tranquil, mind-state (third meaning), which by necessity addresses the relation between an individual and the sonic environment. As such it involves both the concept of situational awareness as well as the definition of soundscape as a relation between an individual and its sonic environment. The link between the state of the environment and ensuing mind-states, in particular the feelings referred to as core affect, has become a recent topic of investigation [2]. Sound annoyed individuals are actually quite explicit about this relation by literally reporting that annoying sounds *force* them out of tranquil mind-states, while pleasant sounds *allow* them to be in tranquil mind-states and therefore allow them *control* over their mind-states [3]. This observation suggests that pleasant sounds play a key-role in both the second and third meaning of quietness. In contrast annoying sounds correspond, via mechanisms described in this paper, to the antithesis of quietness and pleasantness by forcing one out of self-selected mind-states.

While this makes intuitive sense, it requires theoretical cognitive science to integrate concepts like quietness (tranquility), pleasant and annoying sounds, soundscape quality, “audible” safety, core affect, moods, emotions, mind states, focused and divided attention, locus of control, arousal, restoration, and multi-tasking in a single and testable framework that is consistent with available knowledge. As such this paper is an example of inductive science; it starts with a diversity of phenomena and observations and synthesizes these into a few general conclusions. These conclusions should account for the diversity of how sonic environments and soundscapes (as a relation between an individual or society and a sonic environment) can sustain or impede well-being and health.

This paper continues in Section 2 with a selection of factors that influence quietness and annoyance to show that these concepts are closely intertwined with a number of key concepts of cognitive science and perception research. This provides a foundation for Section 3 in which we formulate a detailed and falsifiable model of how sounds, via situational awareness, influence mind-states. In Section 4 we discuss the application of our model for soundscape research. We end with conclusions. 

## 2. A Selection of Relevant Concepts and Knowledge

Many fields and approaches have contributed to a rich and fairly coherent picture of how sounds and sonic environments influence and co-determine our lives. This section outlines a number of key-insights that we will use in Section 3.

### 2.1. Factors Associated with Quietness, Pleasantness, and Annoyance

#### 2.1.1. Holistic Appraisal of the Environment

Quietness (in the second and third meaning) is the result of a holistic assessment of the meaningful relation of the individual to the environment. Recently Booi *et al*. [4] formulated this as follows “*A pleasant sonic environment or soundscape is characterized by the presence of meaningful sounds that concur with the character of the area. The experience of the environment is supplemented by other sensory perceptions: we also see, smell and feel (wind, warmth) the environment. In a tranquil area the total impression is harmonic and no single perception is dominant”.* This formulation of a quiet sense of place entails that models of quietness, by necessity, should rely on a holistic multi-sensory appraisal of all sound sources in context. In addition it suggests that few “foreground” percepts stands out on a “background” of other sounds. 

#### 2.1.2. Quality of Life in Terms of Constrains on Behavioral Options

The definition of annoyance “*as a feeling of displeasure associated with any agent or condition known or believed by an individual or a group to be adversely affecting them*” [5] relies on the experience of someone who is able to “feel” an adverse agent or condition. This definition involves a holistic assessment of the impact of an “agent or condition” to the quality of life of the experiencer. Stansfeld [6] reports that annoyance is related to the belief that one is being avoidably harmed. “*Noise is therefore seen as intrusive into personal privacy, while its meaning for any individual is important in determining whether that person will be annoyed by it*”. Stansfeld concludes: “*The effects of noise are strongest for those outcomes that, like annoyance, can be classified under “quality of life” rather than illness”.* However, a prolonged reduction of quality of life is a health risk. 

Recently, we [7] asked sound annoyed respondents how being sound annoyed influenced their quality of life. The respondents mentioned predominantly reduced options to relax (70%), negative changes in living conditions (68%), less positive or more negative emotions (42%), difficulties attending self-chosen (demanding) activities (15%), and not hearing ambient sounds or difficulties in communicating (6%). The overall response pattern suggests that sound annoyance in the first place corresponds to reduced options to relax and other forms of proactive self-selected behavior. Consistent with the observation that the absence of dominant (attention attracting) percepts is important for quietness, this suggest that annoying sounds become, quite literally, dominant in the sense that they *actively constrain the range of available behavioral options* by constraining mind-states to the here and now. As such one has reduced options to address long-term (health) needs. 

#### 2.1.3. Connecting with the Environment

Evidence concerning the ecological validity of soundscape reproduction [8] suggests that a “feeling of being present”, *i.e.*, being part of the environment, is important for the way we interpret sounds and sonic environments: either objective and detached, as combination of sources, or engaged, in terms of what it means to be part of the sonic environment. This is in accordance with a proposal by de Coensel and Botteldooren [9] that the more we can connect to (a quiet) ambiance, the more we experience feelings of quietness. They propose that “*a feeling of quietness is determined by intervals of silence where silence itself is defined as the ambiance of a soundscape, the gap or distance, the auditory space between sound events”.* The omnidirectional sensitivity of audition makes it ideal to monitor the proximal environment and to warn when something unexpected happens [10], but the more the ambiance is masked, the less our situational awareness can be based on easily available sonic information about the environment. This connection with the proximal environment suggests, again, an important role of background sounds: the more prominent the foreground, the more it masks the background and the less one can connect to the (proximal) environment. 

#### 2.1.4. Sensitivity to Noise

The concept of noise sensitivity also captures the relation between the individual and the environment as essential feature. Job [11] concludes that “*results consistently show that, despite ubiquitous reference to noise sensitivity as a single entity in the literature, in fact noise sensitivity is not a unitary concept. Rather, it generally contains two distinct factors: sensitivity to loud noises produced at a distance from the hearer* (e.g., *road traffic or jackhammer noise*), *and sensitivity to situations of distraction or close but quieter noises* (e.g., *rustling paper at the movies, people talking while watching television*)”. Sensitivity to noise therefore comprises both distal and proximal situational awareness as distinct components, where distal situational awareness is predominantly determined by the loudest (foreground) sounds and proximal situational awareness by the subtle (background) sounds.

#### 2.1.5. Wanted and Unwanted Sound

Noise is unwanted sound, or more precisely: noise is unwanted sound given one’s current needs, goals, and activities. What is unwanted depends on and changes with the history, activities and goals, the physiological state (e.g., fatigue), and in general the needs of the individual. This does not so much entail that the decision of wanted and unwanted sounds differs markedly between individuals. Rather it should be interpreted that it changes *within* individuals as activities, physiological states, and needs develop. In fact Booi *et al*. [4] concludes that “*quietness seems to be a rather universal concept*”, and changing individual needs require a “*diversity in the sonic/acoustic environment. A city can be very noisy, but that is less a problem if its inhabitants have access to quiet places: a quiet home and a quiet place outdoors”.*

#### 2.1.6. The Need for Quietness

Booi *et al*. [4] concludes “*when sound is perceived as a negative factor (noise from transportation and people) there is a higher need for quietness, but as a positive factor (perceived liveliness at home/in neighborhood) it reduces that need*”*.* In addition they observe “*there is also a relation between the need for quietness and the level of education: more highly educated people report a significantly higher need for quietness”.* This suggests that the benefits of an educated mind—presumably via an enhanced control over mind-states and a more pervasive use of directed attention—comes with a higher need for mental restoration (see attention restoration theory below). 

#### 2.1.7. Factors Influencing Soundscape Quality

The literature suggests that soundscape quality often matches perceived quietness. Pheasant *et al*. [12] for example report that “*to achieve a high level of tranquility the percentage of natural features present should be close to 100% and man-made noise sources should be characterized by an LAmax < 55 dB or LAeq < 42 dB. In addition, as the perceived loudness of human and mechanical noise increases the tranquility rating falls, and that as the perceived loudness of biological noise increases so does the tranquility rating”.* This is in complete agreement with the proposal by de Coensel and Botteldooren [9] and matches with reports on soundscape quality. For example Guastavino [13] asked people about their ideal urban soundscape. She reported that the sounds of “other people”, nature, birds, and music were deemed as most pleasurable. On the other hand cars, traffic, and construction work were judged as unpleasant. Within the category “other people” angry people, a small fraction of the neighbors, and cellular phone use were also deemed unpleasant. However in the category “nature”, which comprises wind, water, natural elements, countryside, rain, and parks, none was deemed unpleasant, which is again suggestive of the importance to connect with the (natural) environment. Similarly Nilsson *et al*. [14] reported that the degree to which nature sounds and technological sounds were heard predicted the perceived soundscape quality and noise annoyance. 

This nature/technological sounds trade-off is a common finding [9,14,15,16]. Furthermore, noise annoyance, and reduced soundscape quality “*increases with fear of danger from the noise source, sensitivity to noise, the belief that the authorities can control the noise, awareness of the non-noise impacts of the source and the belief that the noise source is not important*” [17]. In addition Maris *et al*. [18,19] report that fair procedures reduce annoyance. Finally, Yang *et al*. [20] recently concluded, based on EEG-recordings, that visually presented landscape vegetation can “*provide excess noise attenuating effects through subjects’ emotional processing*”*.* All in all it can be concluded that soundscape quality, noise annoyance, as well as the lack of perceived quietness is determined by the fraction of the whole multi-sensory experience that, for a diversity of reasons, demands (immediate) attention.

#### 2.1.8. Relation to Stress and Health

This conclusion dovetails with the recent WHO study [21] which concluded that “*noise is a nonspecific stressor that arouses the autonomous nervous system and the endocrine system which threatens homeostatic or adaptable systems in the body”.* Furthermore Babisch [22] summarizes “*sound/noise is a psychosocial stressor that activate the sympathetic and endocrine system. Acute noise effects do not only occur at high sound levels, but also at relatively low environmental sound levels when, more importantly, certain activities such as concentration, relaxation or sleep are disturbed”.* Both formulations are consistent with the premise of this paper that unwanted sound is unwanted because it reduces options to address one’s needs, which builds up the need for quietness and the associated restorative benefits. Especially, cardiovascular effects are directly related to prolonged (low-level) stress, but also hearing impairments (including tinnitus) result from prolonged exposure to loud sounds. In addition noise causes other effects such as sleep disturbances and reduced work and school performance [21] that can be alleviated or prevented with quietness. 

#### 2.1.9. Role of Home

Quietness is therefore especially important at home [23]. According to Evans *et al*. [24]: “*Home is a place that reflects identity and provides security and maximum control. Good housing offers protection not only from the elements but also from negative social conditions.… Poor housing quality reduces behavioral options, diminishes mastery, and contributes to a general sense of helplessness”.* A good home, and more generally any place we have a positive emotional bond with [25], provides a diversity of options for self-selected adaptive behavior. Poor quality homes and/or neighborhoods reduce these options. In particular sleep patterns [23,26] and relaxation in general are easily influenced by sounds at home and a quiet façade is beneficial [27,28] for quality of life. Like the concept of quietness, the concept of home may refer either to a state of the world or to a state of mind like in “make yourself at home”. which means “make yourself comfortable and at ease”. This entails that a place that “feels like home” is a natural combination of place and tranquility and therefore ideal for restoration.

#### 2.1.10. Attentive Restoration

The concepts of quietness and tranquility may refer to uninterrupted mind-states (third meaning), but not all uninterrupted mind-states are restorative. Attention Restoration Theory (ART) [29] proposes that after prolonged directed attention (e.g., as in task related concentration) it becomes more difficult to suppress exogenous distractions such as noises. Since an attentionally fatigued person is prone to make errors [30] and less able to reach desired (mental) goals easily, he/she experiences irritability [29] and increased arousal. An environment that does not pose any demands on directed attention provides time for the inhibitory mechanisms involved in directed attention to return towards equilibrium. This restores the capacity for direct attention (*i.e.*, control over mind-states) and reduces arousal.

According to ART, four components are important for restoration through suspending direct attention. “*Fascination (use of involuntary, effortless, attention as in direct perception [31]), Being-Away (a physical or cognitive relocation of ones self from everyday obligations), Compatibility (a match between the individual’s desired activity/behavior and the environment) and Extent (the scope and connectedness of the environment in both time and place)*” [32]. Together these (FACE) components ensure prolonged, uninterrupted, and effortless immersion in an environment that is pleasant, self-selected to serve personal needs proactively, and involves minimal directed attention. High quality areas, whether tranquil or lively, provide a range of opportunities for prolonged, uninterrupted, and effortless immersion in a pleasant environment. One’s home should, among other features, facilitate this function. 

#### 2.1.11. Some Sounds Elicit Visceral Responses

While all sudden and loud sounds invoke a startle reflex [33], some sounds are particularly effective in attracting involuntary responses. “*Most people cringe when fingernails are scraped across a chalkboard; for some individuals simply imagining this aversive event evokes a wince*”. [34]. Halpern *et al*. speculate this automatic and almost visceral reaction is associated with warning sounds or with sounds indicative of danger that, regardless the auditory event’s original functional significance, still registers a strong visceral and attention-grabbing response. These types of sounds can even be used as aversive unconditional stimulus (similar to an electric shock) in Pavlovian conditioning in children [35]. The strong aversive and visceral reaction to particular sounds, which promotes conditioning, may be associated with the very cognitive processes that direct attention and which are as such very difficult to ignore. In addition music can increase in heart rate, systolic blood pressure, and stress-related hormones. Again suggesting a close link between sound and visceral responses [36].

#### 2.1.12. Annoying Sounds Disrupt Mind-States

We [3] have shown that 75% of sound annoyed individuals, often literally, mention that the reason why (some) sounds annoy them is because it reminds them involuntarily of the presence of the source while preventing them from maintaining desired mind-states. In addition 25% of the respondents reports that the audibility of disturbing sounds makes it more difficult to relax, sleep, or enjoy their home and garden because they attend sounds they do not want to hear. This entails that these sounds have the capacity to enforce themselves as focus of attention, which in turn entails that these sounds stand out as attended foreground on a background of unattended sounds. It may well be that severely sound annoyed individuals have learned to become perceptual experts in detecting the sounds they do not want to hear. 

#### 2.1.13. Summary

In general it can be stated that all aspects of the relation between individual and environment play roles in appraising quietness, liveliness, and the lack thereof. For quietness there seems to be a key role of, especially natural, background sounds. For pleasantness and annoyance the interplay between fore- and background and the way we appraise these are of central importance. The next subsection addresses appraisal. 

### 2.2. Appraising Sonic Environments

**Figure 1:**
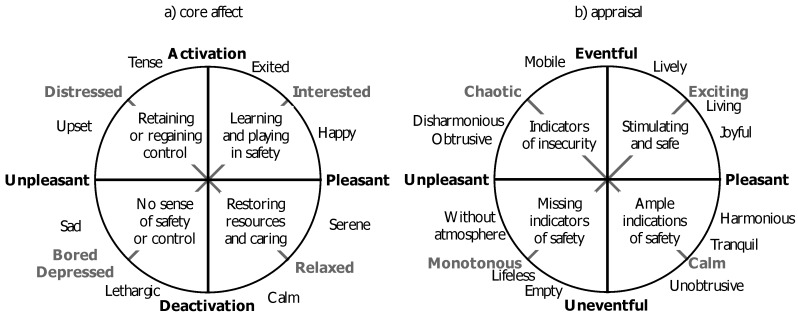
Core affect and appraisal. (**a**) reflects core affect (adapted from [37]), while (**b**) reflects appraisals of the (sonic) environment [15]. The left and right sides of both subfigures are associated with reactive and proactive behavior respectively.

Recently, Kuppens *et al*. [2] reported highly ecologically valid research into the bidirectional relationship between the way we appraise our (current) environment and how that influences how we feel, plan, and act. Kuppens studied this relationship in the context of core affect, which is defined as an integral blend of the dimensions displeasure-pleasure (valence) and passive-active (arousal) [37]. Unlike emotional episodes, which are relatively infrequent, core affect is continually present to self-report. Core affect is usually visualized as a circle with the pleasure axis horizontally and the arousal axis vertically as depicted in Figure 1(a). Here relaxed and invigorated feelings are situated in the lower and upper right quadrants and feelings like boredom and anxiousness in the lower and upper left quadrants respectively. 

The fact that sounds and sonic environments affect behavior entails a direct relation between the way we appraise the situation and the selection of overt behavior. As formulated by Kuppens *et al*. [2], “*appraisals are cognitive evaluations of events that are considered to be the proximal psychological determinants of emotional experience, with different combinations of appraisals corresponding to different emotions*”. According to Kuppens *et al*., appraisals typically refer to (1) motivational relevance (“*Is it important?*”), (2) motivational congruence (“*Is it advantageous or disadvantageous?*”), (3) agency (“*Is it caused by others or myself?*”), (4) problem and emotion focused coping potential (“*Can I cope with the situation and with my emotions?*”), and (5) future expectancy (“*Is the expected outcome desired or not?*”). The first two types of appraisals explicitly refer to motivation, but in fact all five appraisal types refer directly to whether one is free to act or forced to respond: whether one can behave proactively or reactively. 

Core affect influences the interaction with the environment, while the appraisal of the environment influences core affect [2]. It is therefore to be expected that the dimensions that define core affect (as feeling) not only play a role in the study of feelings, but also when appraising visual [38] and sonic environments [15,39,40]. Figure 1(a) shows core affect [37] and Figure 1(b) shows the appraisal of a (sonic) environment [15]. 

Depending on the choice of the researchers, the main appraisal dimensions are either termed pleasantness and eventfulness or a combination of these dimensions rotated by 45°. For example Cain *et al*. [39,41] report the dimensions vibrancy (interpreted as combination between pleasant and eventful) and calmness (combining pleasant and uneventful). Axelsson *et al*. [15] propose to interpret the vibrancy dimension as a continuum from monotonous to exciting and the calmness dimension spanning from calm to chaotic. These two sets of axes at 45° angles are depicted in Figure 1(b). 

As Kuppens *et al*. [2] clearly demonstrate, core affect influences appraisal and appraisal influences core affect. It therefore makes sense to study the adjectives respondents associate with the different parts of the appraisal circumplex [38] to determine what people find important when they appraise sonic environments. We have therefore selected a number of adjectives from Axelsson *et al*. [15] that cover the circle’s circumference. These provide further clues about how to interpret each of the four quadrants and with that the notions of quietness, liveliness, and lack thereof.

The “calm” lower right quadrant opposes the “chaotic” upper left quadrant. According to the New Oxford Dictionary, “calm” is “*the absence of violent or confrontational activity within a place or group*”. The other adjectives in this quadrant support this; unobtrusive: not conspicuous or attracting attention; tranquil: free from disturbance; harmonious: forming a pleasing or consistent whole. In contrast chaotic means “*in a state of complete confusion and disorder*”. Associated words are mobile: able to move or be moved freely or easily; disharmonious: lack of harmony or agreement; obtrusive: noticeable or prominent in an unwelcome or intrusive way. In terms of qualities of the sonic environment it seems “calmness” indicates the absence of events that attract attention because the constituting sounds are part of a consistent and harmonious whole. In contrast “chaotic” seems to indicate the presence of so many conspicuous events that the cognitive capacity to monitor developments of the world is taxed and one is therefore forced to be on constant alert to detect possible (immediate) threats. The calm-chaos axis therefore appears to correspond to the extent the soundscape demands attention and consequently functions as external motivator. 

Upper right opposes lower-left and “exciting” opposes “monotonous”. Exciting means “causing intense and eager enjoyment, interest, or approval to do or to have something”. Monotonous in contrast is defined as “dull, tedious, and repetitious; lacking in variety and interest”. This opposition seems to be indicative of the role of the basic emotion interest [42]. Silva [43] describes interest’s role as to motivate learning and exploration: “by motivating people to learn for its own sake, interest ensures that people will develop a broad set of knowledge, skills, and experience”. And because “one never knows when some new piece of knowledge, new experience, or new friendship may be helpful; interest is thus a counterweight to feelings of uncertainty and anxiety”. The selected adjectives support this. Joyful: feeling, expressing, or causing great pleasure and happiness; living: have an exciting or fulfilling life; lively: (of a place) full of activity and excitement, (of mental activities) intellectually stimulating or perceptive. In contrast “without atmosphere” indicates a place or situation without a pervading tone or mood; “empty”: containing nothing; not filled or occupied; lifeless: lacking vigor, vitality, or excitement. The upper right quadrant is therefore not only exciting, it also represents pleasurable and intellectually stimulating activities that improve personal (interest based) skills, experience, and knowledge and as such provide a sense of fulfillment, vitality, and mastery. In contrast the lower left quadrant is indicative of absences: both of vigor, vitality, excitement, as well as a pervading tone or mood. The monotonous-exciting axis is therefore an intrinsic motivation axis, where the sonic environment reflects few or ample affordances. 

So both diagonal axes represent motivators of behavior. The calm-chaotic axis reflects the degree to which the soundscape functions as *exogenous motivator*: namely to what degree we need to assign mental capacities to the processing and monitoring of the (sonic) environment. The monotonous-exciting axis reflects *endogenous motivation* through the quality and abundance of the affordances offered by the sonic environment. 

These axes can also be interpreted in terms of safety. A chaotic or even threatening environment represents many or strong indicators of actual or potential danger. A monotonous environment may or may not be unsafe; it simply does not offer safety guarantees. This entails that the left halves of Figure 1(a,b) require vigilance. In contrast, a calm environment is a harmonious and congruent whole [44] that provides ample guarantees of safety without sounds that stand out. A calm environment is appraised as one that does not motivate exogenously. And a lively environment provides many interesting sonic affordances in an environment that is safe, interesting, and stimulating; for example through the presence of individuals engaged in self-selected activities only performed in safety. A lively environment may therefore be an environment in which the background is indicative of safety and the (abundant) foreground sounds are both indicative of safety and congruent with current needs and goals. 

Note this argument pertains to (hearing) animals in general. It might be possible to construct a solid case arguing that the close relation between core affect and (soundscape) appraisal in relation to proactive *versus* reactive behavior has existed for hundreds of millions of years. A starting point for such an argument is the *rule of conservative changes* in evolutionary biology. This law states that only those changes can be tolerated, that change essentially nothing [45]. If so, one would expect that the mutual influencing of core affect and appraisal has been preserved since the early evolution of the neural system and as such is associated with (evolutionary old) subcortical processing that can be called *core cognition*. Core cognition determines functions such as the balance between intrinsic and extrinsic motivation, prioritizing (basic) needs [46], fight or flight startle responses [47] *versus* exploration and learning [48], the assignment of attentional resources, and the tuning of arousal [49]. Most of these topics will be addressed in the model of Section 3. 

### 2.3. Summary

We conclude that quietness, liveliness, and the lack thereof are probably easiest understood in terms of how we appraise the sonic environment in terms of safety and opportunities. Different appraisals correspond to different balances between endogenous and exogenous motivation and with that shifting balances between reactive and proactive behavior, which are determined in the process referred to as core cognition. This forms the theoretical basis of the cognitive model introduced in Section 3. 

## 3. Modeling the Influence of Sound on Mind-States

We have formulated a cognitive model describing the cognitive mechanisms through which the sonic environment and the sources that comprise it constrain mind-states. This model explains how quiet and pleasant sonic environments, with ample indicators of safety, allow the listener full freedom and control over mind-states so that short and long term needs can be addressed *proactively*. In contrast, boring sonic environments and sonic environments that contain annoying sounds lack safety indicators or are indicative of potential or actual danger. These environments function as exogenous motivators that force us to be more alert and/or to attend particular sources that then may come to dominate mind-states. Prolonged presence in annoying sonic environments reduces the freedom to self-select adaptive behavior, reduces opportunities for the proactive optimization of long-term needs, and leads to ensuing health effects. 

We developed a first instance of this model [3] to explain how restorative mind-states become progressively more difficult to maintain as task-irrelevant sounds become more and more intruding. The model presented here is an improved and expanded variant that accounts for more phenomena and uses more consistent terminology. It is based on theoretical approaches as diverse as the global workspace theory of consciousness [50], perceptual gist [51], whole-before-details approaches to perception [52,53], Attention Restoration Theory [29], and the cost of interrupted work [54]. 

### 3.1. Overview

A very short summary, to be expanded on in the rest of this section, is that conscious mind-states serve self-selection of adaptive behavior either proactively, to address long-term needs, or reactively, to serve immediate needs. These mind-states need to be based in situational awareness. According to Job [11], situational awareness has two components. One component tracks the overall properties of the environment and relies mainly on the ambiance and the subtle sounds and corresponds to proximal situational awareness. The second component is aimed at specific events within the environment and is typically directed towards the processes that correspond to the loudest (often distal) sounds in the environment. Appraising a situation as safe allows for mind-states for (mental) restoration and proactive adaptive behavior. Diminished safety guarantees, in either the proximal or distal component of noise sensitivity, arouse and lead to mind-states that switch between vigilance and self-selected tasks. If high switching costs, arousal, and vigilance prevent the execution of self-selected tasks: one is dominated by (annoying) sound.

**Figure 2:**
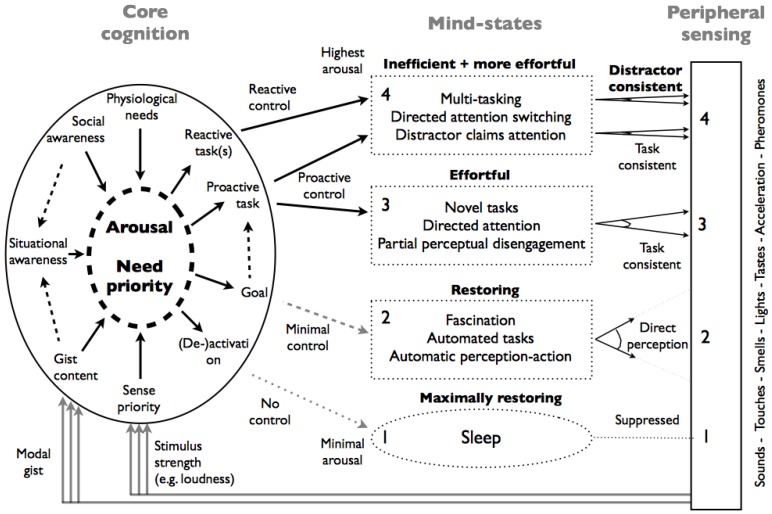
Model of the different processes involved in the experience of quietness and (sound) annoyance. The italicized words in Section 3 refer to this figure.

The italicized terms in Section 3.2 refer to components of the model in Figure 2. The block on the right side depicts the different peripheral sense modalities to be interpreted holistically. The oval on the left denotes the functions of core cognition: tuning arousal, need prioritizing, and task activation. The dotted shapes in the middle reflect four attentional states that, with increasing arousal, are more constrained by core cognition. The lowest corresponds to sleep as the absence of conscious awareness, the second attention capturing by the input in combination with perception-action automatisms, *termed direct perception* [55]. The third and fourth reflect directed attentional states. The fourth state involves effortful switching between proactive and reactive tasks. The words in bold reflect the restorative or effortful quality of the different mind-states. The connecting arrows between the mind-states and peripheral sensing suggest progressively more focused attentional states at higher arousal. 

The proposed model connects (cortical) attentional states with (subcortical) motivational drives as estimated by *core cognition*. The different attentional states correspond to qualitatively different modes of cortical activity: sleep, automated task performance, single task performance, and multi-tasking. While we treat these different modes as separate, they form in actuality a continuum that corresponds to progressively higher arousal and alertness. In fact it seems that arousal and safety (both aspects of core affect) determine which mind-states are accessible. 

The process of allostasis [56], which achieves homeostasis through physiological or behavioral change, is closely associated with *core cognition*: one might interpret core cognition as the continuous strategic decision process of allostasis. If core cognition, with limited and evolutionary old computational capacity, is able to determine that the situation is safe, it allows the cortical workspace freedom to attend proactive self-selection of adaptive behavior—allostasis—in any way, shape, or form it sees fit. But if core cognition is not positively assured of safety, it tasks the cortical workspace, through *arousal*, with a reactive safety assurance, or vigilance, responsibility. An aroused animal or human is: (i) more responsive to sensory stimuli in all modalities; (ii) more active motorically; and (iii) more reactive emotionally [57] and as such better prepared to respond adaptively to the here-and-now.

The model will be addressed in steps. The first step addresses how we can become aware of the environment during wake-up, and maintain that awareness during the rest of the day. The second and third steps address diverse forms of *attentional control* and the relation between perception and attention. The last step addresses the costs of *multi-tasking* and how annoying sounds can prevent the proper execution of self-selected (mental) activities. 

### 3.2. Becoming Aware of the Environment

The first mind-state of a day, *sleep,* is a reversible behavioral state during which one perceptually disengages from the environment. While sleeping, we maintain minimal situational awareness. The disengagement is not absolute, and behaviorally relevant or otherwise salient stimuli of any sensory modality may awaken. With the exception of exhaustion it is not possible to sleep in situations that one deems acutely unsafe, since the associated arousal is prohibitive. 

Everyone can be awakened by a sufficiently loud noise that exceeds the heightened thresholds during sleep. Epidemiological studies [21] show that a quiet (well below 45 dB (A) L_night_) sleep environment is important, since the prevalence of highly sleep-impaired people at 45–49 dB (A) L_night_ is already 4.5%. But loudness is not the whole story: sleepers can wake up from some sounds, while other—potentially louder—sounds will not awake them [26]. Of course sounds are not the only stimuli that can awake us, strong lights, touch, and endogenous stimuli such as a biological clock or physiological needs (like the need to urinate) awake us just as well. 

Waking-up involves a reengagement to the environment through more elaborate *situational awareness*. This reengagement process can be observed as disorientation after one awakes a sleepwalker [58]. The reengagement process is bootstrapped by information about the current place (“Where am I?”) and presumably safety (“Is it safe?”), activities (“What is happening?”), and time (“What time is it?”). Without veridical situational awareness it is impossible to select adaptive behavior (conform the prototypical lunatic who exhibits inappropriate behavior for a given place and time). 

A phenomenon called *perceptual gist* can explain both the selective efficacy of some sounds to wake one up and the bootstrapping of situational awareness. Potter [59] has shown that a preliminary meaningful interpretation of a complex visual scene occurs within only 100 ms after stimulus onset (or sometime after the initiation of a conscious state). This preliminary semantic interpretation is independent of whether or not the scene is expected, it occurs independently of the clutter and the variety of details in the scene, and it contains typically information about where the scene might originate. This fast and preliminary interpretation is called the “gist of a scene” [51] and leads to conscious awareness in the near absence of attention [33]. Harding *et al*. [60] concluded there is ample evidence that auditory processing complies with the ideas initially proposed for vision. Auditory gist content, which hypothesizes an initial meaningful interpretation of the current sonic environment through a crude analysis of ambiance and foreground statistics, can also serve as the starting point of the reengagement process of awaking. The resulting *situational awareness* will be adapted during the whole conscious episode. 

Perceptual analysis appears to be temporally organized from an initial global structuring (based on *gist* and *situational awareness*) towards more and more fine-grained analyses [52]. The properties of visual *gist* [51,61] suggests that visual scenes may initially be processed as a single entity (e.g., a sunny beach), and that segmentation of the scene in objects (e.g., palm trees and tourists) occurs at a later stage. This first gist stage does not require the use of objects as an intermediate representation and does not rely on complex initial stages of perceptual segmentation. However it is also not able to use perceptual details. In the domain of sound the hierarchical decomposition model [53] is similar. In this model, basic stream segregation starts from the whole input (*i.e.*, ambiance and foreground minimally segregated), in which only a single stream can be attended and subdivided further. This form of attention is called selective attention.

Selective attention is a form of *directed attention* that is aimed at the details of one or more perceptual modalities. *Directed attention* is also possible without a direct coupling to perceptual modalities, for example while performing complex calculations or other forms of mental problem solving (as in box 3 of Figure 2). Directed non-perceptual attention requires a decoupling from perceptual input because of its irrelevancy for the particular mental task. This entails a balance between two aspects of directed attention: (perceptual) externally aimed selective attention, which is facilitated by strong or otherwise relevant input, and internally aimed directed attention for mental task performance that requires a precise mental state, unperturbed by perceptual distractors. According to Attention Restoration Theory [62] direct attention involves strong inhibitory processes to suppress undesired mind-states that become progressively more difficult to maintain, and as such create a demand for attentive restoration. 

### 3.3. Attentional Control

The sense whose stimulation awoke the sleeper will dominate the initial (multi-modal) *gist contents* that functions as a seed of situational awareness after wake-up. Situational awareness consists of a crude meaningful interpretation of the here and now on which appropriate coping strategies are based. In addition, situational awareness leads to predicted stimulus statistics that can be compared to gist contents. Mismatches between expected and actual input and particular learned or innate stimulus patterns justify attention shifts towards salient stimuli, conform Carpenter *et al*. [63], to allow conscious analyses of development in the environment. This provides a reliable and continually updated basis for proactive adaptive behavior. 

In accordance with Job’s [11] conclusion that sensitivity to noise involves at least two distinct factors, we propose two stimulus routes through which an initial crude analysis of sensory stimuli exert their influence. Strong unpleasant sensations such as an annoying sound, a foul stench, a burning feeling, and a disgusting taste function as a modality-specific—but within this modality nonspecific—stressor that arouses and motivates to behave *reactively* to re-establish safety. This stimulus route is depicted as *sense priority* in Figure 2 and corresponds to the distal component of sensitivity to noise [11]. The other stimulus route, termed *modal gist*, arouses in a similar way, but on the basis of mismatches between gist contents and situational awareness. 

Attentional control is not only influenced by *stimulus strength* and *gist content* mismatches, also *social needs* (e.g., recognition by others), and *physiological needs* (hunger, thirst) will compete for a fair share of the time attention to be aimed at their particular need. The more we satisfy these needs proactively with, in particular, well-balanced habits “the more our higher powers of mind will be set free for their own proper work” [64].

### 3.4. Direct Perception and Restoration

The form of active perception that is least dependent on attentional control is called *direct perception* [31,55]. In direct perception, depicted as route and box 2, environmental cues guide perception and action. A typical activity based on direct perception is walking on a path. After one has activated a general goal-state, one only has to look in the general direction of the path and somehow the combination of our bodily and perceptual abilities merge perfectly with the route-options afforded by the environment. Direct perception allows sleepwalking and the execution of highly trained automated tasks such as driving home from work. Because direct perception links perception and action inextricably, it is only possible when the environment is sufficiently redundant to guide action. If so, these actions provide additional cues to be processed by the perceptual system, which in turn provides more guidance for the ongoing movement. This loop can persists as long as the conditions for direct perception (e.g., sufficient sensory redundancy) are satisfied. The loop is discontinued when the goal is reached, the need satisfied, or more pressing priorities activated. 

Direct perception (route 2, Figure 2) allows for the existence of stand-alone modules for automatic perception-action relations that only have to be switched on and off and that require minimal control during execution. The main control issue is whether the activity is still relevant as highest priority. This is a situation in which it is desirable for the organism to delegate control to the environment. 

Because direct perception poses minimal demands on directed attentional resources, it requires relatively little mental effort and minimal arousal. A walk in a park is relaxing because it offers opportunities for prolonged, uninterrupted, and effortless immersion in a pleasant environment without posing demands on directed attention (consistent with Attentive Restoration Theory [29]). In addition direct perception plays a central role in habits, which are both activated and controlled by environmental cues and can therefore be activated and executed without conscious control.

### 3.5. The costs of Multi-Tasking

However, depicted as route 4, when particular sounds activate a vigilance task through loudness and gist contents or other influences (situational awareness, social awareness, or physiological needs) they will be prioritized and—at least part of the time—attended to. This then leads to a situation of involuntary multi-tasking and associated task switching, where task-related mind-states have to be partially or completely reconstructed at each switch. At best this leads to an occasional extra mental effort associated with this switching process. At worst it leads to a situation in which the switching costs become so profound that the proper execution of the proactive task becomes impossible: the distractors have become dominant.

Switching cost and the associated arousal may well explain why noise is described as “a nonspecific stressor that arouses the autonomous nervous system and the endocrine system which threatens homeostatic or adaptable systems in the body” [21]. Because the involuntary vigilance task reduces the time spent on proactive tasks, and in general prohibits low arousal states required for restoration, it reduces the time for and quality of restoration and sleep, while at the same time increasing the need for restoration. This explains why sound annoyed (or in general stressed) individuals exhibit a greater need for quietness. So eventually the costs of sound-induced multi-tasking becomes apparent as suboptimal allostasis and eventually disease. 

## 4. Discussion

The previous section introduced a model of the *cognitive and especially the attentive mechanisms* via which sonic environments promote or impede health. In Section 2 we had concluded that the absence of positive indicators of safety force us to attend and address the here and now. Furthermore the availability of (audible) safety indicators allows the freedom of mind to engage in proactive behavior for quality of life and health optimization at all time scales. Although the explicit link between audible safety, proactivity, and freedom of mind-states is new, it is consistent with the extensive literature on quiet areas, sound annoyance, soundscape quality, and restoration as outlined in Section 2. It is also consistent with modern insights in cognitive science that begin to elucidate how conscious states maintain a representation of reality on which adaptive behavior can be based [50]. The concepts that connected the literature analysis in Section 2 with the modeling in Section 3 are core affect, appraisal and core cognition. The two dimensions of core affect, pleasure-displeasure and activated-deactivated, dovetail with vigilance functions which are activated whenever positive indicators of safety cannot be estimated via a cursory (gist-based) analysis of the ambiance and possible foreground sounds. In these situations arousal increases and behavior changes from proactive to reactive, which we, for good reasons, interpret as unpleasant. 

The estimation of positive indications of safety as basic appraisal of the state of the environment is, as far as we know, not yet acknowledged as important in the soundscape literature. Nevertheless, its very basic nature makes it a suitable design guideline. For example in Section 2.2 we coupled the term chaotic to an unpleasant and eventful state of the sonic environment. It is known that a disordered *visual* state of a neighborhood leads to more disorderly and petty criminal behavior [65]. In our terms this might be interpreted that a chaotic/disordered state of affairs, irrespective of the sensory modality, moves a person from proactive behavior, which takes the long term into account (“*It is also my responsibility to keep the neighborhood clean*”), to reactive behavior that addresses only short term needs (“*Everyone litters, so I can as well*”). Designing for positive indicators of safety entails, according to Figure 1, preventing disorder and chaos as much as monotonous and sterile order and promoting environments that our deepest and most basis perceptual processing interpret as either calm or lively (see Figure 1). The growing green movement to reintroduce “nature” in our living environment might do this already and might find additional arguments in this type of reasoning.

Currently our objective measures of sound quality (e.g., loudness, roughness, sharpness) are unconcerned about whether the ambiance is indicative of safety, although these objective measures will reflect certain signal properties indicative of danger. It is important to understand the basis of this relation. In addition we need design tools that allow us to simulate and appraise the resulting soundscapes early in the design process so that the current dominance of visual appraisal can be augmented with auditory appraisal. 

### 4.1. Predictions

**Figure 3:**
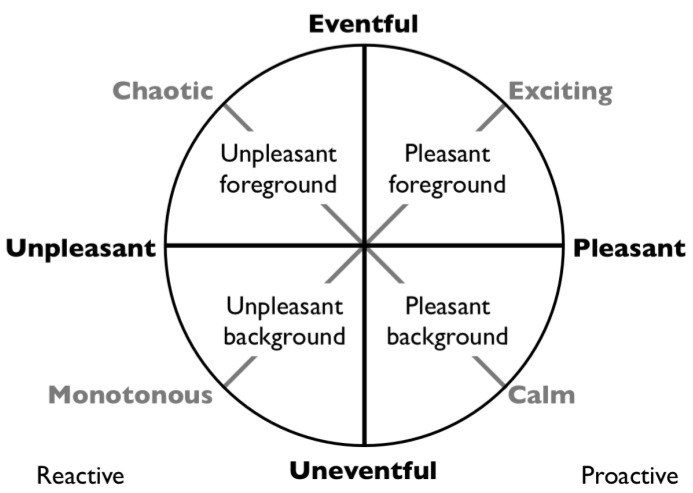
Proposed interpretation of different positions of the core affect circle in terms of fore- and background and pleasant-unpleasant. Unpleasant fore- and background activate a vigilance function and forces one to be more alert, while a combination of a pleasant fore- and background allow full freedom of mind states to attend proactive needs.

The framework presented here leads to a set of predictions that can easily be tested. The literature analysis of quietness and pleasantness in Section 2.1 mentioned the role of fore- and background. The background sounds correspond mainly to proximal situational awareness and to the question “*Is this a safe place?*”. The foreground sounds correspond to the loudest and often more distal events and to the question “*What is going on?*”. For example a calm environment was described in Section 2.1.1 as a harmonious whole without (foreground) sounds that stand out. In combination with the appraisal in Figure 1(b) this suggests that some combination of pleasant and unpleasant fore- and background determines the overall appraisal of a soundscape. This is depicted in Figure 3, where for example a calm environment consists of a pleasant background with few foreground sounds while an exciting or lively environment has many appreciated and well discernible foreground sounds, conform the definition of a HiFi soundscape [66]. A chaotic environment may then be interpreted as a confusing or taxing combination of foreground sounds that on the whole provides no indications of safety (for example because it does not allow one to settle in a stable interpretation of the here and now, see Section 2.2). Finally a monotonous or boring environment has few sounds that stand out on a background not indicative of safety. 

In combination with the two components of sensitivity to noise we interpret the background as the backdrop, constituted by the subtle sounds that define proximal situational awareness, on which foreground activities, determined by the louder sounds of distal situational awareness, are interpreted. If the interpreted background and foreground predict each other and are indicative of safety the result is likely to be interpreted as harmonious. These proposed interpretations can all be tested experimentally. In addition we have proposed that loudness” main role is to prioritize audition over other senses. Which gives it a similar role as stench for smell and pain for touch. 

The model outlined in Figure 2 generates detailed predictions about how quietness and pleasantness can be degraded by activating different functions of core cognition in Figure 2. Conversely quietness/pleasantness can be restored by the introduction of safety indicators. Table 1 lists five possible (sonic) grounds to increase arousal or prevent an elevated level from decreasing. These correspond to the main components on the left of core cognition in Figure 2 and can be tested by a wide variety of methods. 

**Table 1:** Destroying quietness. Core cognition may arouse (cortical) mind-states on qualitatively different grounds. Sounds that act via multiple routes are more arousing and therefore more annoying. Note that the terms in italics correspond to core cognition functions in Figure 2.

Grounds to arouse	Properties
Loudness	Non-specific arousal, associated with reduced range of proximal awareness that prioritizes hearing. This works via the *loudness* route in Figure 2.
Masking of reassuring sounds	The absence or masking of positive indicators of safety that are part of normal natural or social environments. This leads progressively to a need to establish safety actively via conscious processes. Acts via *gist situational awareness*.
Mismatching situational awareness	When expectations about the proximal environment are violated, for example by a (novel) sound that could not be predicted given the current situation. This requires a reorientation of proximal situational awareness. Acts via *gist* if unexpected signal properties are indicative and *situational awareness* (if mismatching semantic properties are indicative).
Explicit indications of danger, typically through source properties	Sonic properties can be indicative of potential danger, this is particularly the case with sounds that elicit negative emotional responses, such as sounds that, if produced by humans (or animals), indicate over-excitation [67]. Acts via *gist* and/or via *social awareness* (indicating distress of unknown others).
Indications of lack of social reassurance	If social sounds are not indicative of safety, they indicate potential danger. They might even be explicitly indicative of conflict or danger as in the case of arguing neighbors. This works via *social awareness* (maybe in combination with *situational awareness* and *gist*).

We propose that the link between sound and health effects is based on prolonged reduction of proactive need satisfaction. This entails that the way people experience their environment in terms of quietness, liveliness, and pleasantness can be used to probe the degree to which they are able to self-select proactive adaptive behavior. The observation of Booi *et al*. [4] that highly educated people appreciate quietness more, suggests the interpretation that they appreciate and/or achieve a higher level of control over their mind-states than people with less education. This has the potential of being developed into in situ measures of cognitive functioning and to predict long-term effects of living in particular environments. 

One of the conclusions of Booi *et al*. [4] was that changing individual needs require a “*diversity in the sonic/acoustic environment*”*.* This suggests that the decision of what is wanted and unwanted sound seems to vary more with changing individual needs than with individual differences. If this conclusion is correct it suggests that a diversity of available acoustic situations (including a sufficient variety of quiet and lively ones) is preferable over a more uniform set of acoustic environments that each comply with some (legal) noise limit. This might lead to considerable monetary savings as well as to more diverse and interesting living environments. 

## 5. Conclusions

This paper addressed the causal link between pleasant and not so pleasant sonic environments and long-term health effects. We argued that the key factor of quiet and lively (*i.e.*, pleasant) environments is the freedom of mind-states they afford through the abundance of safety indications. An analysis of relevant knowledge and an interpretation of core affect in terms of motivation allowed us to formulate a qualitative but detailed cognitive model outlining how the appraisal of sounds can lead to changes in arousal. This leads to reduced access to mind-states associated with long-term optimization of adaptive behavior. As far as we know this is the first detailed cognitive model of the link between sound, appraisal, mind-states, and health. The key role of the switch between reactive and proactive behavior on the basis of audible safety suggests that the (re)introduction of audible indicators of safety into our sonic environments allows people to address their needs better and will therefore be a very effective route towards sustainable health. 
